# Trends and correlates of HIV testing amongst women: lessons learnt from Kenya

**DOI:** 10.4102/phcfm.v5i1.547

**Published:** 2013-09-27

**Authors:** Thomas N.O. Achia, Eunice Obayo

**Affiliations:** 1 School of Mathematics, Statistics and Computer Science, University of KwaZulu Natal, South Africa; 2 School of Public Health, University of the Western Cape, South Africa; 3 School of Mathematics, University of Nairobi, Kenya

## Abstract

**Background:**

A majority of women in Kenya do not know their HIV status and are therefore unable to take preventive measures or medication in order to prolong their lives.

**Objectives:**

This study investigates the key determinants of HIV testing in Kenya and documents how these changed over the 1998–2008 period.

**Method:**

This study uses data from the 1998, 2003 and 2008 Kenya Demographic and Health surveys. Principal components analysis was used to compute indices of HIV knowledge, HIV-related stigma, media exposure and decision making. Survey logistic regression analysis was used to determine factors that had a statistically-significant association with ever having been tested for HIV.

**Results:**

Testing was significantly higher in 2008 compared with the previous surveys. In 1998, 14.7% of the women had tested for HIV. The rate increased to 15.0% in 2003 and then to 59.2% in 2008. In the 1998 and 2003 Kenya Demographic and Health surveys, respondents’ age, region of residence, education, knowledge of someone who had died from HIV-related illness and media exposure were the main determinants of testing. In the 2008 study, HIV-related stigma, occupation and the partner's level of education were found to be associated with HIV testing.

**Conclusion:**

Despite efforts to scale up voluntary counselling and testing in Kenya over the 1998–2008 period, HIV testing amongst women is still quite low. Prevention and control programmes in Kenya need to focus on reducing HIV-related stigma, increasing access to testing in rural areas and increasing access amongst women with little or no education.

## Abstrait

**Tendances et corrélats du dépistage du VIH chez les femmes: l'expérience kenyane**

**Présentation:**

Au Kenya, la plupart des femmes ne connaissent pas leur statut sérologique et ne prennent donc pas de mesures préventives ou traitements pour prolonger la durée de leur vie.

**Objectifs:**

Cette étude révèle les déterminants du dépistage du VIH au Kenya et leur évolution au cours de la période 1998-2008.

**Méthode:**

Cette étude utilise des données issues des Enquêtes démographiques et de santé au Kenya de 1998, 2003 et 2008. Une analyse en composantes principales a été utilisée pour calculer les indices de connaissances sur le VIH, la stigmatisation associée au VIH, l'exposition aux médias et la prise de décisions. Une analyse de régression logistique a été utilisée pour déterminer les principaux facteurs statistiquement associés au dépistage.

**Résultats:**

Le dépistage était plus important en 2008 que dans les enquêtes précédentes. En 1998, 14,7% des femmes s’étaient faites dépister. Un taux qui est passé à 15% en 2003, puis à 59,2% en 2008. Dans les Enquêtes démographiques et de santé de 1998 et 2003, l’âge, le lieu de résidence, l’éducation, le fait d'avoir connu une personne décédée des suites d'une maladie liée au VIH et l'exposition aux médias constituaient les principaux déterminants. Dans l'enquête de 2008, la stigmatisation associée au VIH, la profession et le niveau d’éducation du partenaire étaient les principaux déterminants liés au dépistage.

**Conclusion:**

En dépit d'efforts pour augmenter le conseil volontaire et le dépistage au Kenya au cours de la période 1998-2008, le dépistage du VIH chez les femmes reste relativement faible. Les programmes de prévention et de contrôle au Kenya doivent se concentrer sur la réduction de la stigmatisation liée au VIH, un meilleur accès au dépistage dans les zones rurales et un accès accru chez les femmes ayant fait peu ou pas d’études.

## Introduction

The HIV pandemic is a global concern that has had a profound impact on many aspects of modern society.^1^ By the end of 2011, there were approximately 34 million people living with HIV globally.^2^ The sub-Saharan Africa region was the hardest hit by the pandemic and was home to 69% (23.5 million) of all people living with HIV, accounting for 11%–12% of the global burden.^3^ Women accounted for more than half of all people living with HIV worldwide and nearly 60% of all HIV infections in sub-Saharan Africa.^4^ In Kenya there were approximately 1.6 million people living with HIV, which corresponded to a national prevalence rate of about 7.1%.^5^

Voluntary HIV Counselling and Testing (VCT) has been described as the process by which an individual undergoes confidential counselling in order to enable the individual to make an informed choice about learning his or her HIV status and thereafter take appropriate action.^6^ It is also described as the process whereby individuals or couples undergo pre-test counselling, risk assessment, a same-day rapid HIV test, post-test HIV prevention counselling (often not received in traditional testing) and referral for medical and support services by trained counsellors.^7^ Recent studies have shown that VCT is a cost-effective intervention for reducing HIV-related risk behaviour, particularly when it serves at-risk couples.^8, 9^ It plays a pivotal role in the public-health response to the HIV epidemic and is a vital point of entry to HIV services including primary prevention, prevention of mother-to-child transmission, antiretroviral therapy, management of HIV-related illnesses, tuberculosis control and psychosocial support.^10–12^ VCT also plays a pivotal role in reducing the stigma and discrimination with regard to people living with HIV and is key in the prevention of mother-to-child transmission.^6, 13^

In resource-poor settings, including many sub-Saharan countries with generalised epidemics, VCT is becoming increasingly available, but study results conflict with regard to the potential impact of VCT in promoting a reduction in risky behaviours.^14^

There is evidence to suggest that more than 80% of people living with HIV in Kenya do not know that they are infected.^13^ The Government of Kenya, in partnership with donor organisations, has undertaken an ambitious programme to expand VCT services throughout the country using stand-alone sites and those integrated into other health facilities.^15^ Kenya has had a phenomenal expansion of VCT sites from only three in 2000 to 865 sites in 2007.^16^ Despite the rapid scale-up initiative, the use of VCT services still remains very low.^17, 18^

Several factors have been identified in the literature as determinants of HIV testing. There is, however, no consensus on the main determinants of use. Factors identified include the respondent's age, gender, marital status, education attainment, wealth status, HIV serostatus, area of residence, religion, awareness of a nearby HIV or VCT clinic, belief that everyone should know their HIV status, knowledge of someone with HIV, HIV-related stigma and reported willingness to change sexual behavior if HIV testing was positive, amongst others.^7, 16, 19^

The aim of this study is to identify the main factors that influence HIV testing and the utilisation of VCT amongst women in Kenya. The study also investigated trends of, and patterns in, uptake of VCT over the period 1998 to 2008. We present HIV-testing rates by selected covariates and/or determinants and documents demonstrating how these have evolved over time.

## Methodology

### Data extraction and manipulation

Data on HIV testing and other relevant covariates were extracted from the 1998, 2003 and 2008 Kenya Demographic and Health Surveys (KDHSs). These surveys were designed to provide data that can be used by various stakeholders in order to monitor the population and health situation in Kenya. Data were collected on fertility, family planning and maternal and child health. Other data collected also included HIV prevalence, domestic violence and malaria statistics.

The 1998 KDHS was the third national demographic and health survey conducted in the country. Based on a multistage cluster-sampling approach, a nationally-representative sample of 7881 women aged 15–49 and 3407 men aged 15–54 were interviewed. The 2003 KDHS was implemented using a similar sampling methodology to interview 8195 women aged 15–49 and 3578 men aged 15–54. Finally, the 2008 KDHS was the fifth national demographic and health survey conducted in the country. A total of 9057 households were selected using a multistage cluster-sampling process whereby 8444 women aged 15–49 and 3465 men aged 15–54 were interviewed. The samples in each case provide estimates for Kenya as a whole, for urban and rural areas in each of the eight provinces.

### Ethical considerations

This study was based on secondary data with all participant identifiers removed. Survey procedures and instruments were approved by the Scientific and Ethical Review Committee of the Kenya Medical Research Institute (KEMRI) and by the Ethics Committee of the Opinion Research Corporation Macro International Incorporated (ORC Macro Inc.), Calverton, USA. Ethical permission for use of the data in the present study was obtained from ORC Macro Inc. Details concerning the data-collection protocols are available on the Measures Demographic and Health Surveys (DHS) website (http://www.measuredhs.com/).

### Analysis plan

In this study, we restricted our analysis to women aged 15–49 years and considered the response to the question, ‘Have you ever been tested for HIV?’ to be our primary response variable. Several other variables identified from the literature were cross-classified with this variable. A Pearson's chi-square test was then used to detect any association between the response variable and the categorical variables identified.

Survey logistic-regression analysis, with stepwise elimination, was carried out using STATA 11.0 under the *svy* command and statistically-significant covariates were identified. All *p*-values less than 0.05 were considered to be significant.

Separate statistical analysis was carried out for each of the 1998, 2003 and 2008 data sets.

### Covariates

The independent variables entertained included:

- *Sociodemographic characteristics*: age, region, place of residence, education, occupation, religion, socioeconomic status, marital status, type of marriage, partner's level of education.
- *Socio-cultural factors*: decision making, wealth index, stigma index).
- *Sexual behaviour characteristics*: received gifts for sex in the past 12 months, number of sexual partners in the past 12 months, media exposure, age of first sexual encounter.
- Knowledge and perception characteristics: HIV-risk perception.

Principal component analysis^20^ was used to generate indices regarding HIV knowledge, stigma, media exposure and decision making.

### HIV knowledge-perception index

The HIV knowledge-perception index was created based on responses to the following questions: ‘Can a person reduce risk of getting AIDS (Acquired immune deficiency syndrome) by not having sex at all?’, ‘Can a person reduce the chances of AIDS by always using condoms during sex?’, ‘Can a person reduce the chance of AIDS by having one sex partner with no other partner?’, ‘Can a person get AIDS from mosquito bites?’, ‘Can a person get AIDS by sharing food with person who has AIDS?’ and ‘Can a healthy person have AIDS?’. On the basis of their factor scores, respondents were classified as having low-, moderate- or high knowledge with regard to the causes of HIV and the basic issues surrounding the disease.

### Stigma index

*Stigma* has been defined in the literature as an attribute or label that sets a person apart from others and links the labelled person to undesirable characteristics.^21^ Specifically, stigma related to AIDS has been defined as ‘the prejudice, discounting, discrediting, and discrimination that are directed at people perceived to have AIDS’.^22^ A stigma index was created based on responses to the questions: ‘Willing to care for relative with AIDS’; ‘Person with AIDS allowed to continue teaching’; and ‘Would buy vegetables from vendor with AIDS’. Based on factor scores, respondents were classified as having low-, medium- or high HIV-related stigma.

### Media-exposure index

A media-exposure index was also computed using principal component analysis based on responses to questions posed on the frequency of watching television, listening to radio and reading newspapers. The respondents were then classified as having low-, medium- or high media exposure.

### Decision-making index

The decision-making index was computed based on the respondents’ answers to the questions: ‘Final say on own health care’; ‘Final say on making large household purchases’; ‘Final say on making household purchases for daily needs’; ‘Final say on visits to family or relatives’; ‘Final say on food to be cooked each day’; and ‘Final say on deciding what to do with money husband earns’. The decision-making index was a trichotomous variable with levels ‘independent’, ‘consults’ and ‘subservient’. For the 1998 KDHS, the decision-making index was based only on the response to the question, ‘Who decides how to spend money?’, as the other proxy questions were not included in the survey questionnaire.

## Results

### Summary analysis

Table 1 summarises the results of cross-classifying HIV-testing status with each of the selected covariates. For the 1998 KDHS data set, we found significant bivariate relationships between HIV testing and most of the covariates considered. *Respondent age; Region of residence; Type of place of residence; Level of education; Marital status; Number of other wives;* and *Partner's level of education* were the main sociodemographic factors found to be associated with HIV testing, whilst *Religious affiliation* had no significant association. Most of the other sociocultural, behavioural and HIV knowledge factors, except for the respondent's *Decision-making ability*, *Knowledge of HIV* and *Media exposure*, were associated significantly with HIV testing at the time of the 1998 KDHS.

**TABLE 1:** Design weighted testing rates and *F*-values on cross-classifying HIV testing with selected covariates by year of survey.

Variables	1998 *F* (*p*-value)		2003 *F* (*p*-value)		2008 *F* (*p*-value)	
		
	Never tested	Tested	Never tested	Tested	Never tested	Tested
					
	*Wt*% (*N*)	*Wt*% (*N*)	*Wt*% (*N*)	*Wt*% (*N*)	*Wt*% (*N*)	*Wt*% (*N*)
***Age***	**15.77 (< 0.001)**		**26.96 (< 0.001)**		**82.90 (< 0.001)**	
15–19	90.66 (1542)	9.34 (142)	92.77 (1651)	7.23 (127)	70.39 (1206)	29.61 (533)
20–24	79.70 (1218)	20.3 (287)	80.4 (1358)	19.6 (321)	28.67 (492)	71.33 (1227)
25–29	79.80 (1065)	20.2 (250)	79.82 (1089)	20.18 (284)	23.49 (345)	76.51 (1061)
30–34	83.35 (809)	16.65 (159)	79.04 (868)	20.96 (236)	25.69 (314)	74.31 (854)
35–39	88.64 (863)	11.36 (117)	86.21 (721)	13.79 (124)	36.97 (351)	63.03 (565)
40–44	89.31 (563)	10.69 (71)	90.03 (678)	9.97 (92)	44.81 (323)	55.19 (394)
45–49	90.59 (468)	9.41 (45)	89.65 (446)	10.35 (55)	59.45 (380)	40.55 (281)
***Region***	**18.61 (< 0.001)**		**21.55 (< 0.001)**		**11.89 (< 0.001)**	
Nairobi	69.86 (292)	30.14 (126)	71.48 (814)	28.52 (350)	23.38 (250)	76.62 (690)
Central	79.85 (610)	20.15 (157)	79.54 (1042)	20.46 (269)	41.03 (403)	58.97 (564)
Coast	86.81 (1025)	13.19 (144)	89.46 (828)	10.54 (100)	32.63 (365)	67.37 (778)
Eastern	88.09 (1000)	11.91 (137)	88.18 (869)	11.82 (116)	46.68 (545)	53.32 (570)
Nyanza	88.10 (1186)	11.9 (156)	87.95 (896)	12.05 (127)	36.09 (441)	63.91 (870)
Rift	86.91 (1630)	13.09 (267)	84.69 (1064)	15.31 (175)	44.38 (572)	55.62 (662)
Western	90.21 (785)	9.79 (84)	91.12 (888)	8.88 (99)	43.38 (404)	56.62 (622)
North Eastern	n/c	n/c	99.4 (410)	0.6 (3)	73.42 (431)	26.58 (159)
***Place of residence***	**105.83 (< 0.001)**		**102.53 (< 0.001)**		**58.74 (< 0.001)**	
Urban	74.75 (1101)	25.25 (326)	75.94 (2087)	24.06 (638)	28.33 (793)	71.67 (1794)
Rural	88.49 (5427)	11.51 (745)	88.13 (4724)	11.87 (601)	45.06 (2618)	54.94 (3121)
***Education***	**37.39 (< 0.001)**		**73.36 (< 0.001)**		**20.45 (< 0.001)**	
No	92.73 (881)	7.27 (72)	93.42 (1125)	6.58 (63)	56.47 (693)	43.53 (481)
Primary	87.83 (4006)	12.17 (555)	88.15 (3798)	11.85 (514)	42.21 (1783)	57.79 (2583)
Secondary	78.57 (1544)	21.43 (393)	78.28 (1532)	21.72 (437)	38.44 (789)	61.56 (1286)
Higher	62.43 (97)	37.57 (51)	64.94 (356)	35.06 (225)	20.6 (146)	79.4 (565)
***Religion***	**2.22 (0.0697)**		**5.88 (< 0.001)**		**2.56 (0.0451)**	
Catholic	83.94 (1711)	16.06 (318)	84.89 (1560)	15.11 (311)	42.66 (689)	57.34 (969)
Protestant	85.72 (4200)	14.28 (675)	84.16 (4161)	15.84 (843)	39.44 (1914)	60.56 (3194)
Muslim	83.91 (371)	16.09 (56)	92.51 (930)	7.49 (61)	47.01 (707)	52.99 (614)
None	94.95 (194)	5.05 (10)	89.91 (132)	10.09 (13)	46.21 (77)	53.79 (96)
Other	82.60 (43)	17.4 (9)	79.17 (21)	20.83 (8)	26.64 (18)	73.36 (39)
***Occupation***	**29.78 (< 0.001)**		**49.53 (< 0.001)**		**76.83 (< 0.001)**	
Not working	87.01 (3146)	12.99 (443)	88.82 (2790)	11.18 (341)	50.97 (1882)	49.03 (1788)
Low skill	87.48 (2216)	12.52 (331)	85.76 (2717)	14.24 (465)	39.57 (989)	60.43 (1552)
Highly skilled	77.63 (1157)	22.37 (296)	76.56 (1297)	23.44 (432)	25.81 (530)	74.19 (1565)
***Marital status***	**4.85 (< 0.001)**		**9.12 (< 0.001)**		**62.52 (< 0.001)**	
Never married	88.42 (1949)	11.58 (231)	89.32 (2137)	10.68 (285)	62.27 (1545)	37.73 (964)
Married	84.06 (3859)	15.94 (702)	83.58 (3644)	16.42 (722)	29.98 (1443)	70.02 (3166)
Living together	82.43 (177)	17.57 (36)	80.87 (338)	19.13 (82)	28.59 (109)	71.41 (247)
Widowed	89.05 (260)	10.95 (32)	85.24 (278)	14.76 (51)	45.18 (152)	54.82 (196)
Divorced	81.99 (108)	18.01 (24)	82.41 (120)	17.59 (23)	51.05 (51)	48.95 (66)
Not living together	78.96 (175)	21.04 (46)	80.41 (294)	19.59 (76)	26.72 (111)	73.28 (276)
***No. of other wives***	**4.52 (0.0112)**		**16.49 (< 0.001)**		**10.69 (< 0.001)**	
No other wives	83.18 (3362)	16.82 (646)	81.71 (3175)	18.29 (717)	28.34 (1198)	71.66 (2913)
One other wife	88.85 (480)	11.15 (64)	90.7 (520)	9.3 (57)	60.18 (261)	39.82 (365)
At least two other wives	86.55 (180)	13.4 5(26)	92.74 (193)	7.26 (15)	43.92 (71)	56.08 (78)
***Partners level of education***	**25.56 (< 0.001)**		**32.70 (< 0.001)**		**36.26 (< 0.001)**	
No	92.32 (470)	7.68 (42)	93.13 (729)	6.87 (39)	60.7 (498)	39.3 (300)
Primary	87.71 (2321)	12.29 (333)	87.17 (2135)	12.83 (325)	31.71 (799)	68.29 (1830)
Secondary	79.90 (1554)	20.1 (379)	78.58 (1340)	21.42 (359)	26.51 (448)	73.49 (1290)
Tertiary	65.21 (154)	34.79 (68)	68.43 (392)	31.57 (211)	18.74 (120)	81.26 (528)
Don't know	85.62 (47)	14.38 (7)	82.54 (70)	17.46 (14)	n/c	n/c
***Decision-making ability***	**1.72 (0.1798)**		**41.96 (< 0.001)**		**4.00 (0.019)**	
Independent	82.19 (1451)	17.81 (299)	80.25 (2141)	19.75 (559)	28.74 (464)	71.26 (1083)
Consults	80.12 (717)	19.88 (173)	84.3 (2261)	15.7 (42)	27.56 (469)	72.44 (1301)
Subservient	84.25 (486)	15.75 (86)	90.67 (2405)	9.33 (255)	33.73 (617)	66.27 (1028)
Middle	89.06 (1731)	10.94 (214)	86.7 (1188)	13.3 (180)	44.44 (614)	55.56 (836)
Richer	86.76 (2075)	13.24 (312)	84.74 (1329)	15.26 (235)	41.84 (662)	58.16 (943)
Richest	76.41 (1149)	23.59 (326)	75.36 (1902)	24.64 (641)	27.71 (687)	72.29 (1682)
***HIV-related Stigma index***	**n/c**	**n/c**	**24.14 (< 0.001)**		**45.60 (< 0.001)**	
High stigma	n/c	n/c	89.57 (2434)	10.43 (267)	48.71 (1594)	51.29 (3064)
Moderate stigma	n/c	n/c	82.81 (817)	17.19 (167)	n/c	n/c
Low stigma	n/c	n/c	82.9 (3546)	17.1 (802)	35.45 (1577)	64.55 (1569)
***Age at first sex***	**3.34 (0.0682)**		**43.05 (< 0.001)**		**118.49 (< 0.001)**	
Never had sex	95.80 (1037)	4.2 (42)	95.26 (1292)	4.74 (70)	79.69 (1121)	20.31 (264)
0–10	86.73 (138)	13.27 (17)	94.2 (89)	5.8 (6)	53.67 (47)	46.33 (42)
11–15	86.04 (2227)	13.96 (355)	87.8 (1858)	12.2 (261)	38.46 (717)	61.54 (1246)
16–20	81.89 (2600)	18.11 (529)	81.49 (2819)	18.51 (650)	30.73 (1118)	69.27 (2468)
21–25	77.15 (242)	22.85 (68)	72.81 (410)	27.19 (160)	28.9 (189)	71.1 (460)
26 +	70.62 (25)	29.38 (10)	72.71 (56)	27.29 (25)	19.13 (35)	80.87 (83)
***Sex for gifts***	**8.74 (0.0032)**		**0.28 (0.5952)**		**5.06 (0.025)**	
No	83.94 (5132)	16.06 (948)	82.78 (4370)	17.22 (934)	32.64 (2196)	67.36 (4513)
Yes	78.10 (357)	21.9 (80)	84.12 (255)	15.88 (54)	41.61 (80)	58.39 (130)
***Perceived risk of HIV***	**n/c**	**n/c**	**3.29 (0.0203)**		**41.86 (< 0.001)**	
No	n/c	n/c	86.73 (2617)	13.27 (397)	33.76 (206)	66.24 (409)
Small	n/c	n/c	84.58 (2611)	15.42 (493)	50.04 (1885)	49.96 (1819)
Moderate	n/c	n/c	82.96 (980)	17.04 (218)	34.54 (960)	65.46 (1833)
Great	n/c	n/c	84.08 (588)	15.92 (127)	31.66 (359)	68.34 (854)
***Know someone who has died of HIV***	**20.60 (< 0.001)**		**56.57 (< 0.001)**		**167.93 (< 0.001)**	
No	89.87 (1880)	10.13 (211)	91.33 (1940)	8.67 (178)	57.39 (1218)	42.61 (861)
Yes	83.42 (4541)	16.58 (852)	83 (4847)	17 (1054)	36.43 (2179)	63.57 (4044)
***STI in the last 12 months***	**33.21 (< 0.001)**		**1.15 (0.2844)**		**4.55 (0.0335)**	
No	83.45 (5129)	16.55 (981)	84.98 (6693)	15.02 (1222)	40.85 (3352)	59.15 (4801)
Yes	76.75 (93)	23.25 (26)	88.93 (93)	11.07 (14)	29.41 (41)	70.59 (92)
***Media exposure***	**71.49 (< 0.001)**		**71.51 (< 0.001)**		**16.09 (< 0.001)**	
Low	90.23 (3756)	9.767 (425)	91.81 (2158)	8.19 (168)	45.55 (1794)	54.45 (2020)
Medium	83.65 (1395)	16.35 (271)	86.73 (2630)	13.27 (412)	39.96 (655)	60.04 (1034)
High	75.22 (1265)	24.78 (359)	76.49 (1994)	23.51 (655)	35.08 (955)	64.92 (1853)
***HIV knowledge index***	**0.98 (0.3237)**		**24.15 (< 0.001)**		**24.14 (< 0.001)**	
Low	85.62 (4211)	14.38 (663)	81.26 (3555)	18.74 (894)	46.77 (907)	53.23 (973)
Middle	n/c	n/c	83.77 (478)	16.23 (90)	34.87 (1211)	65.13 (2465)
High	84.64 (2310)	15.36 (407)	89.48 (1664)	10.52 (196)	45.15 (1284)	54.85 (1465)

**TOTAL**	**85.91 (6528)**	**14.09 (1071)**	**85.05 (6811)**	**14.95 (1180)**	**40.79 (3411)**	**59.21 (4915)**

Wt%, weighted testing rates; STI, sexually-transmitted disease; n/c, questions based on these categories were not asked, or no data were available, during the KDHS

For the 2003 KDHS, all the factors considered, except *Gifts for sex* and *Knowledge of HIV*, were associated significantly with HIV testing. The results of the 2008 KDHS differed marginally from the previous two surveys as all the predictors considered were found to be significant.

Table 1 also presents HIV-testing rates by selected covariates for each of the years considered. From an HIV-testing rate of 14.7% in 1998, the rate increased to 15.0% in 2003 and then to 59.2% in 2008. We found no significant difference between the rates of HIV testing in 1998 and 2003. There was, however, a statistically-significant three-fold increase in testing in 2008.

The results indicate an inverted U-shaped relationship between age and testing, with the probability of testing for HIV peaking at age 25 to 29 years for the 1998 and 2008 data sets. The probability of testing peaked at age 20 to 24 years for the 2003 data set.

Testing rates varied significantly by region for the three years considered. In all three years, testing rates were highest in Nairobi, the capital city of Kenya. A comparison between testing rates in 2003 and 2008 suggests differentials in percentage improvement in testing rates. Coast province, with a 57% improvement, had the highest percentage improvement in testing rate, followed by Nyanza and Nairobi provinces with 52% and 48% improvement, respectively. North Eastern province had the lowest testing rates and also the lowest percentage improvement in testing for both 2003 and 2008.

The results suggested higher testing rates in urban areas compared with rural areas for all three periods. A comparison of the 2003 and 2008 HIV-testing rates suggests that there was a greater percentage improvement in testing rates for urban areas (47.6%) compared with rural areas (43.1%). The rates of HIV testing increased linearly with the respondent's level of education, socioeconomic status and partner's level of education for each of the years. The highest percentage increase in testing was observed in wealthier or better-educated women.

Amongst the behavioural determinants considered, a positive relationship was found between HIV-testing rate and age of first sexual encounter. The nature of the relationship between HIV-testing status and women receiving sex for gifts differed for each year considered. In 1998, the rate of HIV testing was higher amongst women receiving gifts for sex (21.9%) as compared with those who had not (16.1%). In the next time periods, significantly-higher testing rates were found -amongst low-risk women (i.e. those who did not accept gifts for sex).

### Logistic regression results

Table 2 presents the results of the logistic-regression models fitted to each of the three datasets. It is apparent from these results that key drivers of HIV testing had, to a large extent, changed over time.

**TABLE 2:** Logistic regression modelling of HIV testing by determinants of HIV testing.

Variables	1998		2003		2008	
		
	OR (95% CI)	AOR (95% CI)	OR (95% CI)	AOR (95% CI)	OR (95% CI)	AOR (95% CI)
**Age**						
15–19	1.00	1.00	1.00	1.00	1.00	1.00
20–24	2.47 (1.92,3.18)	2.33 (1.80,3.02)	3.13 (2.46,3.98)	2.54 (1.97,3.26)	5.92 (4.52,7.73)	1.24 (0.69,2.21)
25–29	2.46 (1.93,3.13)	2.32 (1.79,3.01)	3.25 (2.50,4.21)	2.74 (2.10,3.58)	7.74 (5.78,10.37)	1.02 (0.61,1.70)
30–34	1.94 (1.43,2.63)	1.87 (1.35,2.59)	3.41 (2.65,4.38)	2.91 (2.25,3.76)	6.88 (5.11,9.25)	0.78 (0.46,1.30)
35–39	1.24 (0.89,1.73)	1.35 (0.95,1.91)	2.05 (1.53,2.76)	1.75 (1.29,2.37)	4.05 (2.98,5.51)	0.45 (0.26,0.77)
40–44	1.16 (0.81,1.66)	1.40 (0.97,2.02)	1.42 (1.03,1.97)	1.26 (0.91,1.74)	2.93 (2.11,4.07)	0.30 (0.18,0.53)
45–49	1.01 (0.66,1.53)	1.27 (0.83,1.96)	1.48 (1.02,2.16)	1.39 (0.93,2.07)	1.62 (1.22,2.16)	0.19 (0.11,0.33)
**Region**						
Nairobi	1.00	1.00	1.00	1.00	1.00	1.00
Central	0.58 (0.42,0.81)	0.82 (0.59,1.13)	0.64 (0.52,0.79)	0.92 (0.72,1.17)	0.44 (0.33,0.58)	0.57 (0.36,0.91)
Coast	0.35 (0.25,0.50)	0.53 (0.38,0.74)	0.30 (0.20,0.43)	0.54 (0.39,0.74)	0.63 (0.47,0.85)	0.90 (0.52,1.55)
Eastern	0.31 (0.22,0.44)	0.48 (0.35,0.67)	0.34 (0.25,0.45)	0.65 (0.47,0.90)	0.35 (0.26,0.46)	0.51 (0.32,0.80)
Nyanza	0.31 (0.23,0.44)	0.54 (0.39,0.75)	0.34 (0.25,0.47)	0.71 (0.51,0.99)	0.54 (0.40,0.73)	0.61 (0.38,0.99)
Rift	0.35 (0.26,0.47)	0.50 (0.38,0.66)	0.45 (0.34,0.60)	0.82 (0.62,1.09)	0.38 (0.27,0.54)	0.51 (0.30,0.86)
Western	0.25 (0.17,0.37)	0.40 (0.28,0.58)	0.24 (0.18,0.33)	0.48 (0.35,0.66)	0.40 (0.29,0.54)	0.65 (0.39,1.08)
North Eastern	n/c	n/c	0.02 (0.00,0.05)	0.08 (0.03,0.25)	0.11 (0.07,0.18)	0.24 (0.12,0.46)
**Place of residence**						
Urban	1.00	n/c	1.00	n/c	1.00	n/c
Rural	0.38 (0.32;0.46)	n/c	0.42 (0.36;0.50)	n/c	0.48 (0.40;0.58)	n/c
**Education**						
No	1.00	1.00	1.00	1.00	1.00	n/c
Primary	1.77 (1.25;2.49)	1.30 (0.89;1.89)	1.91 (1.36;2.68)	1.12 (0.78;1.60)	1.78 (1.38;2.28)	n/c
Secondary	3.48 (2.42;5.00)	1.81 (1.20;2.73)	3.94 (2.79;5.57)	1.65 (1.12;2.42)	2.08 (1.50;2.87)	n/c
Higher	7.67 (4.32;13.64)	2.85 (1.56;5.21)	7.67 (5.13;11.46)	2.19 (1.40;3.41)	5.00 (3.55;7.04)	n/c
**Religion**						
Catholic	1.00	n/c	1.00	n/c	1.00	n/c
Protestant	0.87(0.73,1.03)	n/c	1.06(0.89,1.26)	n/c	1.14(0.99,1.32)	n/c
Muslim	1.00(0.66,1.52)	n/c	0.45(0.32,0.65)	n/c	0.84(0.63,1.12)	n/c
None	0.28(0.12,0.63)	n/c	0.63(0.30,1.31)	n/c	0.87(0.53,1.41)	n/c
Other	1.10(0.42,2.89)	n/c	1.48(0.45,4.84)	n/c	2.05(0.89,4.72)	n/c
**Wealth index**						
Poorest	1.00	n/c	1.00	1.00	1.00	1.00
Poorer	1.60 (1.01,2.53)	n/c	1.73 (1.22,2.43)	1.47 (1.05,2.06)	1.21 (0.99,1.48)	1.00 (0.76,1.32)
Middle	1.41 (0.89,2.24)	n/c	2.37 (1.70,3.30)	1.80 (1.30,2.51)	1.29 (1.07,1.56)	1.05 (0.79,1.40)
Richer	1.76 (1.11,2.77)	n/c	2.78 (1.98,3.90)	1.71 (1.20,2.44)	1.44 (1.17,1.76)	0.93 (0.69,1.26)
Richest	3.55 (2.24,5.63)	n/c	5.05 (3.67,6.93)	2.21 (1.53,3.18)	2.70 (2.17,3.35)	1.73 (1.07,2.80)
**Marital status**						
Marital status	1.00	n/c	1.00	n/c	1.00	n/c
Married	1.45 (1.19,1.76)	n/c	1.64 (1.39,1.95)	n/c	3.85 (3.24,4.59)	n/c
Living together	1.63 (1.05,2.52)	n/c	1.98 (1.47,2.65)	n/c	4.12 (2.81,6.04)	n/c
Widowed	0.94 (0.58,1.51)	n/c	1.45 (1.02,2.07)	n/c	2.00 (1.43,2.79)	n/c
Divorced	1.68 (0.97,2.90)	n/c	1.79 (1.04,3.05)	n/c	1.58 (0.84,2.97)	n/c
Not living together	2.03 (1.32,3.14)	n/c	2.04 (1.48,2.81)	n/c	4.53 (2.99,6.85)	n/c
**No of other wives**						
No other wives	1.00	n/c	1.00	n/c	1.00	n/c
One other	0.62 (0.45,0.86)	n/c	0.46 (0.33,0.64)	n/c	0.60 (0.46,0.78)	n/c
At least two other wives	0.77 (0.48,1.24)	n/c	0.35 (0.19,0.66)	n/c	0.51 (0.31,0.83)	n/c
**Partners level of education**						
No	1.00	n/c	1.00	n/c	1.00	1.00
Primary	1.68 (1.11,2.55)	n/c	1.99 (1.30,3.05)	n/c	3.33 (2.59,4.27)	1.72 (1.33,2.21)
Secondary	3.02 (1.97,4.63)	n/c	3.69 (2.42,5.64)	n/c	4.28 (3.10,5.91)	1.79 (1.32,2.43)
Tertiary	6.41 (3.80,10.83)	n/c	6.25 (4.03,9.71)	n/c	6.70 (4.70,9.54)	2.15 (1.49,3.10)
Don't know	2.02 (0.78,5.20)	n/c	2.87 (1.34,6.14)	n/c	n/c	n/c
**STI in the last 12 months**						
No	1.00	n/c	1.00	n/c	1.00	n/c
Yes	1.53 (0.97,2.42)	n/c	0.70 (0.37,1.34)	n/c	1.66 (1.04,2.65)	n/c
**Know someone who has died of HIV**						
No	1.00	n/c	1.00	n/c	1.00	n/c
Yes	1.76 (1.45,2.14)	n/c	2.16 (1.76,2.65)	n/c	2.35 (2.06,2.68)	n/c
**Sex for gifts**						
No	1.00	n/c	n/c	n/c	n/c	n/c
Yes	1.47 (1.14,1.89)	n/c	0.91 (0.63,1.30)	n/c	0.68 (0.48,0.95)	n/c
**Media exposure**						
Low	1.00	1.00	1.00	1.00	1.00	n/c
Medium	1.81 (1.49,2.19)	1.51 (1.23,1.85)	1.72 (1.36,2.17)	1.23 (0.97,1.56)	1.26 (1.06,1.48)	n/c
High	3.04 (2.51,3.70)	2.02 (1.63,2.50)	3.45 (2.73,4.35)	1.45 (1.13,1.86)	1.55 (1.31,1.83)	n/c
**HIV-related Stigma index**						
High stigma	n/c	n/c	1.00	n/c	1.00	1.00
Moderate stigma	n/c	n/c	1.78 (1.42,2.24)	n/c	n/c	n/c
Low stigma	n/c	n/c	1.77 (1.48,2.12)	n/c	1.73 (1.47,2.03)	1.52 (1.23,1.86)
**HIV knowledge index**						
Low	1.00	n/c	1.00	n/c	1.00	n/c
Middle	n/c	n/c	0.84 (0.64,1.10)	n/c	1.64 (1.39,1.93)	n/c
High	1.08 (0.93,1.26)	n/c	0.51 (0.42,0.62)	n/c	1.07 (0.91,1.25)	n/c
**Occupation**						
Not working	1.00	n/c	1.00	n/c	1.00	1.00
Low skill	0.96 (0.80,1.16)	n/c	1.32 (1.11,1.56)	n/c	1.59 (1.35,1.87)	0.99 (0.78,1.26)
Highly skilled	1.93 (1.60,2.33)	n/c	2.43 (2.03,2.91)	n/c	2.99 (2.54,3.52)	1.44 (1.13,1.83)
**Perceived risk of HIV**						
No	n/c	n/c	1.00	n/c	1.00	n/c
Small	n/c	n/c	1.19 (1.02,1.39)	n/c	0.51 (0.39,0.66)	n/c
Moderate	n/c	n/c	1.34 (1.10,1.64)	n/c	0.97 (0.76,1.22)	n/c
Great	n/c	n/c	1.24 (0.97,1.58)	n/c	1.10 (0.84,1.43)	n/c
**Decision making**						
Independent	1.00	n/c	1.00	n/c	1.00	n/c
Consults	1.15 (0.92,1.42)	n/c	0.76 (0.64,0.89)	n/c	1.06 (0.86,1.31)	n/c
Subservient	0.86 (0.65,1.15)	n/c	0.42 (0.35,0.50)	n/c	0.79 (0.63,1.00)	n/c
**Age of first sex**						
Never had sex	1.00	n/c	1.00	n/c	1.00	n/c
0–10	3.49 (1.79,6.82)	n/c	1.24 (0.46,3.31)	n/c	3.39 (2.01,5.71)	n/c
11–15	3.70 (2.55,5.38)	n/c	2.79 (2.06,3.78)	n/c	6.28 (4.93,7.99)	n/c
16–20	5.05 (3.53,7.21)	n/c	4.57 (3.48,5.99)	n/c	8.84 (7.07,11.06)	n/c
21–25	6.76 (4.32,10.57)	n/c	7.51 (5.62,10.03)	n/c	9.65 (6.92,13.47)	n/c
26 +	9.49 (3.77,23.93)	n/c	7.54 (4.42,12.86)	n/c	16.59 (8.90,30.90)	n/c

OR, Odds Ratio; AOR, Adjusted Odds Ratio; CI, Confidence Interval; STI, sexually-transmitted disease, n/c, variables were omitted for the best fitting model and so adjusted odds were not computed; 1.00, reference data

In 1998, the principal determinants of testing were the respondent's *Age, Region of residence, Education* and *Media exposure*. Adjusting for other covariates, the odds of testing for HIV testing amongst individuals aged 15–19 years was approximately half that of individuals in each of the age categories 20–24, 25–29 and 30–34. The odds of testing for individuals above the age of 35 years were not different significantly from the odds for individuals in the 15–19 year age-group. We also noticed on adjusting the other predictors that chances of testing for HIV were higher in Nairobi compared with the other seven provinces (except Central province). Furthermore, the odds of testing for women with moderate media exposure were 1.5 times higher than for women with low media exposure. The odds of testing among women with high media exposure was more than double that for women with low media exposure.

In addition to the four factors found to explain HIV testing in 1998, we also identified *Socioeconomic status* and *Knowledge of a person who had died of AIDS* to be significant in 2003. The results indicate an inverted U-shaped relationship between *Age* and testing. This phenomenon was observed for all three surveys. HIV testing amongst respondents in the 20–24, 25–29 and 30–34 year age categories was significantly higher, approximately triple, the rate in the 15–19 group. The rate of testing was 75% higher in the 35–39 category compared with the 15–19 year age group.

We also noted, adjusting for other covariates, significant regional differentials in 2003. The rates of testing were respectively 95%, 52%, 46%, 35% and 29% lower in North Eastern, Western, Coast, Eastern and Nyanza provinces. The rates of HIV testing in the other two provinces (Central and Rift Valley) were not different significantly from the rates in Nairobi. Adjusting for the effects of other covariates, knowing a person who died from HIV-related illness increased the chances of testing by 70% and 72% in 2003 and 2008, respectively. In the 2008 survey we also noted a 52% higher chance of HIV testing amongst individuals with lower HIV-related stigma compared with those with high stigma.

In contrast with the previous two surveys, we found (adjusting for other covariates) a decline in HIV testing with age in 2008. The rates of testing in the 20–24, 25–29 and 30–34 age groups were not different significantly from the testing rates in the 15–19 year age group. The testing rates, however, declined by 55%, 70% and 81% in the 35–39, 40–44 and 45–49 year age groups, respectively. Unlike the previous survey (2003), we found significantly-lower rates of testing in Central and Rift Valley provinces as compared with Nairobi province, as was also the case in other regions in the country.

## Trustworthiness

As detailed in the methods section of this study, the study design and methods used to obtain the data in this study are sound scientifically. For each of the three years, multistage cluster-sampling procedures were used to select the clusters, households and hence the women included in this study. The results presented in this study can therefore be generalised to all women aged 15–49 years living in Kenya during the study period. The scales used in this study have been pretested and used in other studies. The reliability of all the indices developed was assessed using the Cronbach's alpha coefficients and thereafter used to evaluate their impact on VCT use.

## Discussion

In this study, we described patterns and trends in HIV testing in Kenya over the period of 1998 to 2008. Our database consisted of national surveys conducted in 1998, 2003, and 2008. It is apparent from this study that the proportion of women being tested for HIV had increased significantly over time. Although only marginal improvement was seen over the 1998 to 2003 period, a marked increase in the testing, from 15.0% in 2003 to 59.2% in 2008, was noted. This could possibly be attributed to government initiatives to expand VCT services throughout the country using stand-alone sites and those integrated into other health facilities.^15^ Despite this improvement in access to VCT services, as well as the implementation of wide-ranging interventions by both government and non-government organisations in Kenya, HIV prevalence over the period 1998 to 2008 remained relatively high and unchanging.^23^ A large proportion of people living with HIV were unaware of their HIV status,^24^ posing a major challenge in preventing HIV transmission and providing effective care to infected persons.

We also set out to interrogate the main sociodemographic, sociocultural, behavioral and HIV knowledge-based determinants of HIV testing and to determine whether the effects of these drivers remained the same over time. In the first two surveys (KDHS-1998 and KDHS-2003), we found sociodemographic characteristics such as age, region of residence and respondent level of education, alongside socioeconomic status, media exposure and the knowledge of someone who had died of HIV-related illness to be the main determinants in a woman's decisions to test for HIV. In the subsequent survey, a woman's occupation, level of HIV-related stigma and her partner's level of education also played an important role in HIV testing.

Various socioeconomic variables have been shown to be important in explaining HIV testing within the sub-Saharan Africa context.^25–30^ Studies suggest that the poor and less educated are less likely to test for HIV. Our findings are consistent with these studies.^25, 30–32^ In line with other studies in literature,^31, 33^ we also found that women employed in highly-skilled jobs were more likely to test for HIV than those who were in lower-skilled jobs or unemployed. Highly-skilled employment possibly alludes to better education and better access to information, thereby granting the women access to sound information on HIV and the importance of VCT. We also found a strong association between HIV testing and the partner's level of education. The probability of testing was found to increase with an increased partner level of education.

We also found higher HIV testing rates in urban areas compared with the rural areas. As suggested elsewhere,^25^ these variations could possibly be attributed to lower access to testing centres in the rural areas. Our results suggested improved access to HIV testing services over time, with a larger percentage improvement occurring in urban areas.

We found a significant regional variation in testing rates. Testing rates in Nyanza province were still quite low despite the fact that the region has an HIV prevalence of 14.9% (double the national average) and carries 30% of the national burden.^34^

Religion has also been identified as a key determinant of HIV testing in many other settings.^25, 30, 35^ Despite the documented evidence of differences between Muslims and those with no religious affiliation,^36^ we found no significant difference in HIV-testing rates between the two groups. These low testing rates by Muslims have been linked to adherence to religious tenets that give protection against sexually-transmitted HIV.^29^ Adjusting for other covariates, however, we found no association between religion and HIV testing across all three surveys.

In this study we also interrogated the relationships between HIV testing and three constructs (HIV-related stigma, HIV knowledge and media exposure) that have been suggested in other settings. In line with previous studies within sub-Saharan Africa,^28, 29, 33, 36–45^ our unadjusted results suggested a strong and inverse association between HIV-related stigma and HIV testing. The impact on HIV testing as a result of knowing someone who had died of an HIV-related illness has been mentioned by various authors.^25, 30, 46^ Results from this study showed that knowing someone who has died of an HIV-related illness increased the chances of testing for HIV by more than 70% for both the 2003 and 2008 surveys. There are several other studies, however, that have suggested that such knowledge has no impact on the HIV testing.^47^ Studies also suggest that depending on the relationship with the individual who has died from HIV-related illness and the potential stigma attached to HIV infection, one may not want to know one's HIV status.

## Limitations

In this study, information about HIV-testing status was based on self-reported responses. This could potentially be affected by recall bias if the test was offered a long time ago and could also be affected by the tendency of respondents preferring to give socially-desirable answers. Measurement of stigma, a key variable, may not have been satisfactory due to insufficient questions to cover the all the possible dimensions of HIV-related stigma. It is assumed that participants in each of the surveys were independent of each other and that the responses to the questions on testing were also independent. Our analysis did not account for any spatial heterogeneity or autocorrelation that may have been present in the data.

## Conclusion

This study used cross-sectional data from the 1998, 2003 and 2008 Kenya Demographic and Health Surveys to determine trends and patterns in VCT uptake in Kenya. We found a marked improvement in testing rates over the last five years but noted that HIV testing amongst women is still quite low.The study has revealed a socioeconomic differential in HIV testing rates and indicates the need to aim health promotion programmes at women of lower income, education and employment cadres. It is therefore necessary to encourage and support the use of VCT, alongside programmes that encourage behavioural change to reduce the incidence of HIV. HIV prevention and control programmes in Kenya need to focus on reducing HIV-related stigma, increasing access to testing in rural area and on programmes that increase access amongst those with little or no education.
